# Factors associated with the utilization of antenatal care and prevention of mother-to-child HIV transmission services in Ethiopia: applying a count regression model

**DOI:** 10.1186/s12905-018-0679-9

**Published:** 2018-11-19

**Authors:** Elias Asfaw Zegeye, Josue Mbonigaba, Zacharie Tsala Dimbuene

**Affiliations:** 1 School of Accounting, Economics and Finance, Economics Department, University of KwaZulu-Natal, Durban, South Africa; 2 Clinton Health Access Initiative, Health Care Financing Program, Addis Ababa, Ethiopia; 3 African Population and Health Research Center, Statistics and Survey Unit, Nairobi, Kenya

**Keywords:** PMTCT service, Ethiopia, Urban-rural settings, Count regression, Utilization analysis

## Abstract

**Background:**

Prevention of Mother-to-Child HIV Transmission (PMTCT) coverage has been low in Ethiopia and the service has been implemented in a fragmented manner. Solutions to this problem have mainly been sought on the supply-side in the form of improved management and allocation of limited resources. However, this approach largely ignores the demand-side factors associated with low PMTCT coverage in the country. The study assesses the factors associated with the utilization of PMTCT services taking into consideration counts of visits to antenatal care (ANC) services in urban high-HIV prevalence and rural low-HIV prevalence settings in Ethiopia.

**Methods:**

A multivariate regression model was employed to identify significant factors associated with PMTCT service utilization. Poisson and negative binomial regression models were applied, considering the number of ANC visits as a dependent variable. The explanatory variables were age; educational status; type of occupation; decision-making power in the household; living in proximity to educated people; a neighborhood with good welfare services; location (urban high-HIV prevalence and rural low-HIV prevalence); transportation accessibility; walking distance (in minutes); and household income status. The alpha dispersion test (a) was performed to measure the goodness-of-fit of the model. Significant results were reported at *p*-values of < 0.05 and < 0.001.

**Results:**

Household income, socio-economic setting (urban high-HIV prevalence and rural low-HIV prevalence) and walking distance (in minutes) had a statistically significant relationship with the number of ANC visits by pregnant women (*p* < 0.05). A pregnant woman from an urban high-HIV prevalence setting would be expected to make 34% more ANC visits (counts) than her rural low-HIV prevalence counterparts (p < 0.05). Holding other variables constant, a unit increase in household income would increase the expected ANC visits by 0.004%. An increase in walking distance by a unit (a minute) would decrease the number of ANC visits by 0.001(*p* < 0.001).

**Conclusion:**

Long walking distance, low household income and living in a rural setting are the significant factors associated with low PMTCT service utilization. The primary strategies for a holistic policy to improve ANC/PMTCT utilization should thus include improving the geographical accessibility of ANC/PMTCT services, expanding household welfare and paying more attention to remote rural areas.

**Electronic supplementary material:**

The online version of this article (10.1186/s12905-018-0679-9) contains supplementary material, which is available to authorized users.

## Background

Globally, 36.7 (34.0–39.8) million people were living with HIV/AIDS, 2.1 (1.8–2.4) million were newly infected with HIV, and about 1.1 (940,000–1.3) million people had died of HIV/AIDS in 2015 [1]. Over time, an increasing number of people living with HIV, and decreases in new HIV infections and AIDS-related deaths have been reported [1–3]. For instance, new HIV infections in 2015 were 5% lower than in 2010 [1], while they were 20% lower in 2011 than in 2001 [2]. UNAIDS further reported a decrease of 33% in HIV infections between 2001 and 2012 [3].

The overall decline in new infections and deaths can be partly attributed to the effectiveness of prevention, behavioral changes and treatment interventions [1, 4, 5]. However, low uptake of interventions targeting pregnant women and infants in low- and middle-income countries (LMICs) persists. According to UNAIDS, approximately 38% of HIV-exposed pregnant women and 65% of HIV-infected infants, mainly in LMICs, have no access to antiretroviral therapy (ART) services [3]. A minimal improvement from 64 to 68% Prevention of Mother-to-Child HIV/AIDS Transmission (PMTCT) coverage was reported during the period 2012–2013 [6] and this increased to 77% in 2015 (UNAIDS, 2016). The situation was worse in sub-Saharan African (SSA) countries where PMTCT services were underutilized and weak [2, 7]. In the 21 priority countries, AIDS-related mortality (< 15 years) decreased by 53% between 2009 and 2015 [1].

Low levels of service uptake among pregnant women attending antenatal care (ANC) are a critical bottleneck in PMTCT interventions as these integrated services are a primary entry point for comprehensive PMTCT service. For instance in 2015, only 34% of pregnant women accessed lifelong antiretroviral treatment [6]. Some countries also reported a decrease in PMTCT service utilization between 2012 and 2013 [6]. PMTCT service uptake was uneven due to differences between health systems and fragile due to social factors [6–8]. Limited utilization has important implications given the fact that PMTCT is at the center of health improvement among the most vulnerable segment of the population, notably women and children.

A systematic review revealed that utilization of PMTCT services was hampered by many challenges including patient-related factors, socio-cultural barriers, negative family influence, poor adherence, inadequate diagnostic laboratory facilities and a lack of effective community participation (Adetokunboh and Oluwasanu, 2015). Thus, despite the cross-cutting role of PMTCT in reducing new infant infections and prolonging women’s life expectancy, utilization of these services has been inadequate. These points to the need for improved PMTCT program management, particularly in low-income countries like Ethiopia that carry a substantial portion of the pandemic burden.

### PMTCT service utilization

The few studies that have analyzed PMTCT service utilization in Africa, including in South Africa, Uganda and Burkina Faso [9–14] reported different barriers to effective PMTCT service uptake. These include health facility inaccessibility, long distances, poor infrastructure, poverty, inadequate knowledge of PMTCT, service quality, limited family and community support, inaccessible rural communities and partners’ influence. Adetokunboh and Oluwasanu highlighted the need for more information on health service utilization to achieve the elimination of mother-to-child HIV transmission. Overall, demand-side PMTCT service utilization has received inadequate research attention in SSA countries [8].

Utilization of PMTCT services has also been at the center of policy debates. Extending the vision of the Joint United Nations Program on HIV/AIDS (UNAIDS) to achieve zero infant infections by 2015 [3, 15], the United Nations (UN) Sustainable Development Goals (SDG) adopted in September 2015 set the goal of a major reduction in maternal and child deaths as well as universal access to reproductive health services by 2030 [16]. Given growing income inequalities, PMTCT service utilization is expected to vary across urban-rural settings due to enabling factors on the one hand and barriers on the other, related in particular to service accessibility and improving coverage. However, only a few studies are reported in South Africa and Uganda, which assessed program implementation across different socio-economic, structural and urban-rural settings [11, 17].

As in most SSA countries, there is limited evidence on the utilization of PMTCT services in Ethiopia. This problem is significant as the disease has become more heterogeneous across urban-rural settings in this country [18], and service uptake is likely to be low in remote rural settings as compared to urban areas. Different confounding factors such as knowledge, educational attainment, and partner influence and facility accessibility have also negatively affected rural women [19, 20].

PMTCT services in Ethiopia are characterized by low coverage [21]. This led UNAIDS to identify it as one of the 21 countries that require high-priority action [3]. In 2012, UNAIDS noted that Ethiopia had achieved less than 50% ART coverage for pregnant women living with HIV [3]. The 2011/12 Ministry of Health Annual Performance Report noted that a total of 9775 HIV-positive mothers had received ART treatment in the 2004 financial year (Ethiopian Fiscal Year/EFY), far below the target (38,405) [2]. Coverage increased from 9.3% of the target in 2010/11 to 25.5% in 2011/12, indicating unsatisfactory progress [22].

However, none of the above studies assessed PMTCT program utilization within the context of urban-rural HIV heterogeneity (high, low) settings. Given that this epidemic is heterogenous across urban-rural settings, it is worth generating evidence (applying regression models) to enable policy makers to take different factors into account, such as those that are patient related and those associated with demographics and socio-economic circumstances (urban high-HIV prevalence vis-à-vis rural low-HIV prevalence settings) that affect service utilization. Such an analysis should inform overall PMTCT program implementation and lead to improved service coverage across the country. To this end, this study assessed the factors associated with PMTCT service utilization using data on pregnant women from ANC/PMTCT integrated services in Ethiopian health care facilities.

## Methods

### Study settings

The selection of participating health facilities in this study was based on the 2012 ANC sentinel surveillance of PMTCT by the Ethiopian Public Health Institute (EPHI) [23, 24]. The EPHI identified 117 ANC facility sites in different geographical regions in the country and found that HIV prevalence in these facilities ranged from 0.01 to 17%. Six facilities with the highest HIV prevalence (between 8.1 and 17%) and six with the lowest HIV prevalence (< 0.1%) were purposively enrolled. The choice of highest and lowest HIV prevalence health facilities (ANC-PMTCT sites) was informed by the need to obtain a clear and comprehensive picture of the factors associated with the utilization of ANC-PMTCT services across high HIV prevalence versus low prevalence areas. Given that urban settings have higher HIV prevalence, the six facilities with the highest prevalence were from urban areas, and the six with the lowest prevalence were in rural settings. This offered an opportunity to assess the effect of existing interventions in urban high-HIV prevalence/rural low-HIV prevalence areas from the perspective of PMTCT service utilization. The selected health facilities are in Amhara, South Nation and Nationality People (SNNP), Oromia, Harrar, Dire Dawa and Addis Ababa regions, where 85% of the country’s population resides [25]. Table 1 describes the facilities included in the study.

**Table 1:** List of study sites, including HIV prevalence and location of health facility

Surveyed health facilities	Region	HIV prevalence	Region	Settings
Bahir Dar Hospital	Amhara	17.30	Northern Ethiopia	Urban high-HIV prevalence
Hiwot Fana Hospital	Harar	8.82	Eastern Ethiopia	Urban high-HIV prevalence
Dile Chora Hospital	Dire Dawa	8.12	Eastern Ethiopia	Urban high-HIV prevalence
AFRTH Hospital	Addis Ababa	8.70	Addis Ababa	Urban high-HIV prevalence
Soddo Health Center	SNNPR	8.81	Southern Ethiopia	Urban high-HIV prevalence
Teklehaimanot Health Center	Addis Ababa	8.83	Addis Ababa	Urban high-HIV prevalence
Limuseka Health Center	Oromia	0.02	Western Ethiopia	Rural low-HIV prevalence
Daddim Health Center	Oromia	0.04	Western Ethiopia	Rural low-HIV prevalence
Toke Health Center	Oromia	0.01	Western Ethiopia	Rural low-HIV prevalence
Chewaka Health Center	Oromia	0.05	Western Ethiopia	Rural low-HIV prevalence
Kokosa Health Center	Oromia	0.02	Eastern Ethiopia	Rural low-HIV prevalence
Hasange Health Center	Harar	0.03	Eastern Ethiopia	Rural low-HIV prevalence

*Source: ANC sentinel HIV/AIDS PMTCT surveillance report (EPHI, 2014)*

### Sample size estimation

The study population for the survey comprised of pregnant women attending ANC/PMTCT services at the surveyed health facilities. As the PMTCT service is well-integrated with ANC in these facilities, utilization evidence was collected from pregnant women attending the ANC service. As noted previously, ANC is the primary entry point for the PMTCT service package that comprises pre-HIV test counseling, testing, post-test counseling and enrollment in lifelong antiretroviral treatment. Every pregnant woman attending ANC would be expected to utilize the PMTCT service package. With this framework, the sample for ANC/PMTCT service utilization was computed using a single proportion formula, 95% confidence interval, 5% margin of error, the proportion of 42% of HIV-positive tested women and a non-response rate of 10%, and assuming a homogenous population of pregnant women attending the health facilities [26, 27]. Applying the formula, the minimum required sample size was calculated as 449.

A relatively large sample of 484 pregnant women was used to analyze the factors associated with demand-side utilization of PMTCT services. These women were accessed through simple random sampling of past ANC/PMTCT records of visits (from the registration book) across the 12 health facilities. Using the visit records, the total sample size was proportionally apportioned across the surveyed health facilities. Based on the inclusion criteria, every pregnant woman reporting for ANC/PMTCT service visits during the study period had an equal chance of being selected.

Women attending other reproductive health services, including family planning were not included. If the pregnant woman had come with her partner (husband) to the health facility, conditional on her consent, the husband would be included in the interview but the response was finally recorded for the pregnant woman. Once the respondents agreed, data collection was conducted in an appropriately designated and confidential space within the health facility premises, assisted by a trained data collector. Data collectors were trained over two days on the data instruments and relevant data variables. If a pregnant woman withdrew during the course of the interview, she would be replaced by the next, consecutive randomly sampled pregnant woman. This process was repeated until the required proportionally allocated sample limit was reached per health facility.

The data was collected using a structured questionnaire, which was adapted from a similar ANC survey conducted in Ethiopia [28], and piloted in two health facilities in Addis Ababa, namely, Gandhi Memorial Hospital and Beletsheachew Health Center. The questionnaire consisted of sections on socio-demographic characteristics; information about the visit; HIV/AIDS counseling and testing; socio-economic status; out-of-pocket (OOP) spending; household income and spending, and related socio-cultural factors. It was translated back into the national language, Amharic.

### Regression model

**Fig. 1:**
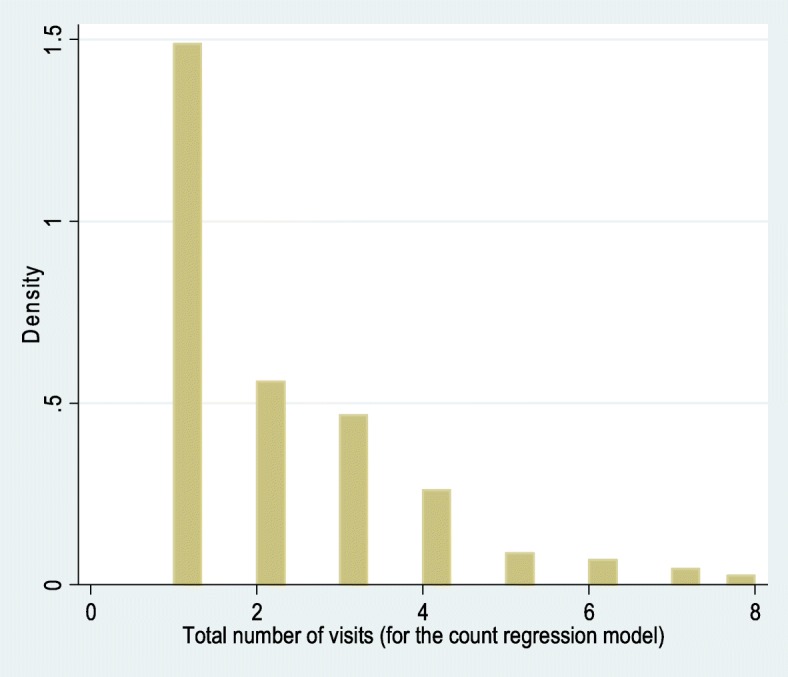
Histogram showing the density of the number of antenatal care visits (for the count regression model)

The count model was applied to analyze the frequencies/counts of the pregnant women’s ANC visits (including first through to eighth visits) at the health facilities. The minimum and maximum recorded visits (during the survey) ranged from one to eight, respectively. The dependent variable was the reported number of visits for ANC initiation or follow-up. The basic assumption was that a pregnant woman with a large number of visits (counts) during her pregnancy would be more likely to use the PMTCT service. Because the number of ANC visits was considered as the dependent variable (Y) in the model, count regression (Poisson and negative binomial) models were the most appropriate to assess the statistically significant factors associated with PMTCT service utilization. The distribution of the ANC visits and mean and variance of the dependent variable is presented in Table 2 and Fig. 1.

**Table 2:** Mean and variance of the dependent variable (number of ANC visits by the pregnant women)

Total number of visits (for the count regression model)				
	Percentiles	Smallest		
1%	1	1		
5	1	1		
10%	1	1	obs	482
25%	1	1	Sum of Wgt.	482
50%	2		Mean	2.134855
		Largest	Std. Dev.	1.508282
75%	3	8		
90%	4	8	Variance	2.274916
95%	5	8	Skewness	1.5466144
99%	7	8	Kurtosis	5.233386

As shown in Fig. 1, the data was skewed to the right, which calls for application of the count model, either the Poisson or negative binomial model. The relevant statistical analysis software was applied to estimate the mean and variance demands for the appropriateness of the count model analysis. The mean count variable was 2.13, which was similar to the variance result (2.27). If the mean and variance of the dependent variables are equal, the Poisson regression model would best fit the dependent variable of interest (i.e., the number of ANC visits by a pregnant woman) [29].

The Poisson model determined the probability that the regressed variable (Y) occurs at a specific time interval. The probability is modeled as:

1.1 $$ \rho\ \left(\ Y=y\right)=\frac{e^{-\mu }{\mu}^y}{y!} $$

1.2 $$ \mu =\exp \left({x}_i\beta \right) $$

Where,

Y_i_ = number of pregnant women attending ANC visits (1st, 2nd, 3rd… 8th);

μ = the intensity or rate parameter;

Xi = are the explanatory variables.

The variables represented by Xi in Eq. 1.2, were age; educational status; type of occupation; decision-making power in the household; living in proximity to educated people; a neighborhood with good welfare services; location (urban high-HIV prevalence, rural low-HIV prevalence); transportation accessibility; walking distance (hours/minutes); and household income status. In addition, the dummy variable on the socio-economic location and HIV prevalence setting variable were used jointly (urban high-HIV prevalence vis-à-vis rural low-HIV prevalence) as a relevant explanatory variable. Some of the independent variables were continuous, while others were categorical (dummy) variables.

The second count regression model was the negative binomial model. It is similar to the count regression model but with a different functional form and the *equidispersion* property (mean and variance equality) in the Poisson model is relaxed. The negative binomial model was primarily applied to strengthen the findings from the Poisson regression model, and estimate the alpha dispersion (α) tests. The negative binomial model, which allows the variance and the mean not to be equal, was written as follows:

1.3 $$ \mathit{\operatorname{var}}\ \left(y|x\right)=\upmu +\upalpha\ {\mu}^2 $$

This model also tests (α) the “*measure of over dispersion”* and was used to strengthen the choice of the Poisson model. According to the null hypothesis (H_o_: α = 0 or H_a_: ≠0), tests whether α is significantly different from zero can yield three different outcomes: 1) if α = 0, no dispersion (this shows the appropriateness of the Poisson model), 2) if α > 0, over dispersion and 3) if α < 0, under dispersion.

## Results

Of the 484 pregnant women attending the health facilities who were interviewed, 49% were from urban high-HIV prevalence settings, while 51% lived in rural low-HIV prevalence areas. The mean age of the respondents was 27 and 25 years in the urban and rural settings, respectively. The majority of the respondents in urban high-HIV prevalence settings (more than 57%) had completed secondary school (grades 9 & 10) or above, while most of the rural low-HIV prevalence interviewees (more than 86%) were illiterate. A statistically significant difference in terms of educational attainment was thus found between women from urban high-HIV prevalence and rural low-HIV prevalence areas attending ANC (*p* < 0.001). In both settings, most of the pregnant women did not have a formal occupation, and worked as housewives. The respondents depended on their partner/husband and his sources of income. A relatively higher proportion of pregnant women in urban high-HIV prevalence settings had permanent paid jobs (25%) and they were involved in trade or business-related activities (17%). However, in the rural low-HIV prevalence settings, most women worked in farming and agriculture. Permanent paid work in the urban high-HIV prevalence areas and farming and agriculture in the rural low-HIV prevalence settings, were the second most important types of occupation (*p* < 0.001). The detailed socio-demographic characteristics of the respondents are presented in Table 3.

**Table 3:** Socio-demographic characteristics of the respondents (pregnant women attending ANC services) across urban high-HIV prevalence and rural low-HIV prevalence settings

Socio-Demographic profiles	Urban high-HIV prevalence setting *n* (%)	Rural low-HIV prevalence setting *n* (%)	*P*-value
Age	27.2 (SD = 5.00)	25.91 (SD = 5.54)	0.051
Educational Status			0.000^†^
Illiterate	21 (14.4)	125 (85.6)	
Basic Education/ read & write	23 (54.8)	19 (45.2)	
Primary (grade 1–8)	57 (50.4)	56 (49.6)	
Secondary (grade 9–10)	43 (71.7)	17 (28.3)	
Preparatory (11–12)	22 (75.9)	7 (24.1)	
10 + TVET	11 (68.8)	5 (31.3)	
College/university - Diploma	31 (67.4)	15 (32.6)	
College/university - Degree	26 (83.9)	5 (16.1)	
College/university - Masters	1 (100)	0 (0.0)	
Occupation			0.000^†^
Permanent paid employee	60 (71.4)	24 (28.6)	
Wage Laborer	7 (53.8)	6 (46.2)	
Farming/ agriculture	6 (10.2)	53 (89.8)	
Trade/business/Merchant/self-employed	40 (64.5)	22 (35.5)	
Housewife	102 (42.5)	138 (57.5)	
Housemaid	9 (81.8)	2 (18.2)	
Student	11 (73.3)	4 (26.7)	
Marital Status			0.027^††^
Single	5 (100)	0 (0.0)	
Married	214 (47.5)	237 (52.5)	
Divorced/Separated	9 (81.8)	2 (18.2)	
Widowed	2 (33.3)	4 (66.7)	
Live with partner/cohabiting	5 (45.5)	6 (54.5)	

^†^Statistical significance at *P* < 0.001

^††^Statistical significance at *P* < 0.05

### Characteristics of ANC/ PMTCT services visits

Of all the study participants, 58 and 42% attended the health facilities for their first pregnancy in the urban high-HIV prevalence and rural low-HIV prevalence settings, respectively. In both settings, most of the respondents were attending their first to fourth ANC visits. A pregnant woman in an urban high-HIV prevalence setting made a relatively higher number of ANC visits than one in a rural low-HIV prevalence area (*p* < 0.05). The detailed characteristics of the respondents’ use of ANC/PMTCT services are shown in Table 4. Based on this descriptive analysis, the number of visits was considered as the dependent variable in the count regression model.

**Table 4:** Characteristics of the respondents accessing ANC/ PMTCT services across the urban high-HIV prevalence and rural low-HIV prevalence settings

Key variables	Urban high-HIV prevalence settings *n* (%)	Rural low-HIV prevalence settings *n* (%)	*P*-value
Mean months of pregnancy	7.6 (SD = 13.72)	7.4 (SD = 10.27)	0.105
Is this your first pregnancy?			0.001^†^
Yes	98 (58.3)	70 (41.7)	
No	136 (43.2)	179 (56.8)	
If NO, indicate the total number of pregnancies you have had including this one			0.178
Two	43 (45.3)	52 (54.7)	
Three	49 (55.1)	40 (44.9)	
Four	18 (32.1)	38 (67.9)	
Five	11 (35.5)	20 (64.5)	
Six and more	15 (34.1)	29 (65.9)	
Is this your first antenatal visit (1 ANC) for this pregnancy (at any health facility visited)?			0.009
Yes	98 (42.2)	134 (57.8)	
No	136 (54.2)	115 (45.8)	
Is this your first antenatal (1 ANC) visit at this facility for this pregnancy?			0.003^†^
Yes	100 (41.7)	140 (58.3)	
No	134 (55.1)	109 (44.9)	
If NO, please tell us the total number of ANC visits you have had including this one			0.001^†^
Two	44 (48.9)	46 (51.1)	
Three	36 (48.6)	38 (51.4)	
Four	21 (50.0)	21 (50.0)	
Five	11 (78.6)	3 (21.4)	
Six and more	22 (100.0)	0 (0.0)	

^†^Statistical significance at *P* < 0.05

### Regression analysis

Table 5 shows the results from the Poisson regression model analysis. The model estimation applied vce (robust) to make the error estimation more robust and control for minor violations related to the underlying assumptions [30]. Household income, socio-economic HIV prevalence setting (urban high-HIV prevalence, rural low HIV-prevalence) and walking distance (minutes) had a statistically significant relationship with the number of ANC visits by a pregnant woman (Y) (*p* < 0.05). The Poisson regression estimates that a pregnant woman from an urban high-HIV prevalence setting would be expected to make 34% more ANC visits than her rural low-HIV prevalence counterpart. In other words, if the pregnant woman lives in a rural low-HIV prevalence setting, she would be expected to make fewer ANC visits than a woman residing in an urban high-HIV prevalence area (*p* < 0.05). Holding other variables constant, a unit increase in household income would increase the expected ANC visits by 0.004%. An increase in household income was thus a statistically significant factor in relation to an increase in ANC visits (*p* < 0.001). However, if the walking distance increases by a unit (a minute) the expected number of ANC visits would decrease by 0.001. Increased walking distance was significantly associated with a lower expected number of ANC visits (*p* < 0.05).

**Table 5:** Poisson regression model result (estimate of incidence rate ratio) applying the robust error estimation

Poisson, irr		Number of obs = 220		
		Wald chi2 (11) = 88.54		
Poisson regression		Prob > chi2 = 0.00		
		Pseudo R2 = 0.07		
Log pseudolikelihood = −348.13				
Visits	IRR	P > |z|	[95% Conf. Interval]	
Age	1.01	0.36	0.99	1.02
Education *(literate compared to illiterate)*	1.01	0.92	0.80	1.28
Occupation *(non-farm occupation compared to housewife)*	0.96	0.71	0.78	1.18
Occupation *(farm/agriculture compared to housewife)*	0.99	0.89	0.80	1.22
Proximity to educated people *(yes, no)*	0.94	0.57	0.78	1.15
Proximity to neighborhood with good welfare services *(yes, no)*	1.09	0.40	0.89	1.34
Transport access *(yes, no)*	1.03	0.77	0.83	1.29
Walking distance *(minutes/hours)*	0.10	0.02	0.10	0.99
Income	1.00	0.00	1.00	1.00
Urban-rural settings *(urban high-HIV prevalence compared to rural low-HIV prevalence)*	1.40	0.00	1.14	1.73
Balanced decision-making *(yes, no)*	0.95	0.60	0.79	1.14
Cons	1.58	0.05	1.01	2.48

However, model variables, such as the age of the respondent; educational status; type of occupation; proximity to educated people; a neighborhood with better living standards/good welfare services; and transport accessibility were not found to be significantly related to a pregnant woman’s expected number of visits to health facilities. The model was run to estimate the incidence rate ratio (IRR), which measures the rate at which the event (ANC visits) occurs. Table 5 illustrates the IRRs estimated across the explanatory variables.

The significant explanatory variables were household income, urban-rural HIV prevalence settings and walking distance, which had IRRs of 1.00004, 1.4023 and 0.9991, respectively (Table 4). Holding other factors constant, the percentage increase in the incidence rate of ANC visits was 0.004% for every unit increase in income. The incidence rate (of ANC visits) for urban high-HIV prevalence dwellers is 1.40 times that for the reference group (i.e., pregnant women in rural low-HIV prevalence settings), ceteris paribus. In addition, the percentage change in the incidence rate of ANC visits was a 1% decrease for every unit (minute) increase in walking distance.

### Statistical tests for the model

The negative binomial model was analyzed to cross-validate the results in Table 5 and for the alpha dispersion test (a). The reported coefficient was similar in both the negative binomial and Poisson regression models. This highlights the appropriateness of the Poisson regression model estimation in eqs. 1.1–1.4 and further strengthens the statistical variables estimated for the dependent variable (i.e., the number of visits by a pregnant woman). The likelihood ratio test of alpha (a) (measure of dispersion) was zero, which informs the appropriateness of the Poisson model as a good-fit for the datasets. Moreover, the chi-square test (that measures two degrees of freedom for the exogenous variable) applied to occupation found that the variables were not statistically significant (Tables 6 and 7). The detailed results from the negative binomial regression are explained in (Additional file 1).

**Table 6:** Two degrees of freedom of chi-square test for explanatory variable ‘occupation’ (a: negative binomial and b: Poisson model)

. test 1. Occupation 2. Occupation		. test 1. Occupation 2. Occupation	
(1)	[Visits] 1. Occupation = 0	(1)	[Visits] 1. Occupation = 0
(2)	[Visits] 2. Occupation = 0	(2)	[Visits] 2. Occupation = 0
	chi2 (2) = 0.12		chi2 (2) = 0.14
	Prob > chi2 = 0.9431		Prob > chi2 = 0.9314
	*(a: negative binomial)*		*(b: Poisson) model*

**Table 7:** Statistical test *(estat gof)* for the Poisson model function

estat gof	
Deviance goodness-of-fit = 137.38	
Prob > chi2 (208) = 1.00	
Pearson goodness-of-fit = 151.97	
Prob > chi2 (208) = 0.99	

Applying the “estat gof” function (i.e. the goodness of fit tests for Pearson regression models), the model also showed the goodness-of-fit chi-square measure. Table 6 shows that the Pearson goodness-of-fit was not statistically significant; this indicates that the model did fit the datasets well. Moreover, the dispersion test alpha (a), and mean and variance were equal, which substantiates and validates the model results using the Poisson regression model.

## Discussion

According to the Poisson regression model, a pregnant woman from an urban high-HIV prevalence setting would be expected to make more ANC visits (counts) than her rural low-HIV prevalence counterpart. A pregnant woman residing in a rural low-HIV prevalence setting would be expected to make fewer ANC visits compared to a woman residing in an urban high-HIV prevalence setting (*p* < 0.05). Other studies have also indicated the limited effectiveness of PMTCT services in rural remote areas [9, 12, 13] and have highlighted the relevance of socio-economic (urban-rural) factors in explaining PMTCT service uptake [9, 31]. Malaju and Alene’s study in north-western Ethiopia found that PMTCT service acceptability/utilization patterns were associated with the number of ANC visits made by pregnant women residing in urban versus rural settings [19, 32].

Household income is also significantly associated with a higher number of ANC visits, and hence contributes to improved PMTCT service uptake. Various studies support this finding. For instance, a systematic review of 28 studies in developing countries noted that household economic factors and women’s employment are critical factors in determining the use of ANC services [33]. Family income and women’s employment status also play a significant role in the uptake of overall maternal and child health services [34, 35].

Other studies have reported that, from the patient perspective, economic circumstances and household income are determinants of the utilization of maternal and child health care services. Newacheck and Kim examined the effect of OOP expenses on service utilization, particularly for special health needs [36]. Out-of-pocket expenses (for service fees and transportation) incurred by a pregnant woman or her household have been found to influence service utilization among those attending ANC [37, 38]. Given the lack of social health insurance schemes in SSA, OOP spending accounts for one- to two-thirds of a patient’s total health expenditure [39]. The most recent Ethiopian National Health Account reported that OOP spending accounted for 33% of total spending in the sector [40], which possibly affects the overall health sector utilization pattern. In sum, OOP expenses affect household consumption, and have been found to be a deterrent to utilization of health care, including PMTCT services [41].

The model analysis also highlights the significant relationship between walking distance to a health facility and PMTCT service utilization. PMTCT utilization studies in South Africa confirm this finding [9–11]. Skinner et al. analyzed PMTCT service utilization in the Eastern Cape town of Flagstaff [9], which is one of the poorest and most underdeveloped communities in South Africa. The qualitative findings from in-depth interviews and focus group discussions showed that long travelling distances and inaccessible transportation facilities were the two key barriers to utilizing PMTCT services [9]. A similar study in rural Mali that focused on the factors determining maternal health service utilization [42] also reported that service inaccessibility and transportation problems were critical barriers.

We did not find any statistically significant relationship between PMTCT service utilization and age; educational status; type of occupation; decision-making in the household; living in proximity to educated people; a neighborhood with good welfare services, and transportation accessibility. However, other studies have reported that most of these factors impact on PMTCT service uptake [34, 43]. While they used a different method employing qualitative analysis techniques, Peltzer et al. (2007) also found that transportation or geographical inaccessibility, limited family or community support, and communication facilities were critical deterrents to effective PMTCT service utilization [10, 11].

Although this analysis highlighted the critical factors that impact on the utilization of PMTCT services, the study has important limitations. In order to accommodate urban-rural HIV heterogeneity, the health facilities were selected purposively and this may affect the national representativeness of our findings. In addition, due to the limited availability of data, additional potential explanatory variables, namely, quality of health care, adherence and loss to follow-up, and availability of trained healthcare providers were not considered in the regression model, which probably alters the findings. We strongly recommend that both these demand and supply-side factors should be considered in future regression model analysis.

## Conclusion

In contrast to most developed countries, PMTCT programs have been poorly implemented in a fragmented manner in limited-resource SSA countries. In Ethiopia in particular, antiretroviral treatment coverage for HIV-positive pregnant women and their infants was less than 50%. In light of the current universal health coverage agenda, strategies to address this situation and increase the uptake of PMTCT services include women’s economic empowerment, according high priority to rural low-HIV prevalence settings, and enhancing transportation accessibility. Health policy makers and program implementers need to take these three factors into account in seeking to expand PMTCT service packages for those in need, and to achieve the 90–90-90 UNAIDS target in limited resource settings. On the other hand, the real impact of these factors (urban-rural HIV prevalence, income status and waking distance) on the HIV epidemic transmission needs to be assessed in the future.

## Additional file

Additional file 1: Negative binomial regression analysis results on the number of visits by the pregnant women. (DOCX 18 kb)

## Abbreviations

ANC: Antenatal care
EFY: Ethiopian fiscal year
EPHI: Ethiopian Public Health Institute
IRR: Incidence rate ratio
LMICs: Low- and middle-income countries
OOP: Out-of-pocket
PMTCT: Prevention of Mother-to-Child HIV Transmission
SDG: Sustainable development goals
SNNP: South Nation and nationality people
SSA: Sub-Saharan Africa
UN: United Nations
UNAIDS: Joint United Nations Program on HIV/AIDS
