# Supplementary material for: Factors associated with the utilization of antenatal care and prevention of mother-to-child HIV transmission services in Ethiopia: applying a count regression model
Source: BMC Womens Health. 2018 Nov 19;18:187. doi: 10.1186/s12905-018-0679-9 (PMC6245866; doi:10.1186/s12905-018-0679-9)
Supplement: Supplementary file 1 — Negative binomial regression analysis results on the number of visits by the pregnant women. (DOCX 18 kb) [file 12905_2018_679_MOESM1_ESM.docx]

Additional file 1

Table 1: Negative binomial regression analysis results on the number of visits by the pregnant women

| Negative binomial regression | |  |  |  |  |  |  |
| --- | --- | --- | --- | --- | --- | --- | --- |
|  |  |  |  |  | Number of obs = 220 | | |
|  |  |  |  |  | LR chi2 (11) = 50.12 | | |
| Dispersion = mean | |  |  |  | Prob > chi2 = 0.00 | | |
| Log likelihood = -348.13 | |  |  |  | Pseudo R2 = 0.067 | | |
|  |  |  |  |  |  |  |  |
| **Visits** | | **Coef.** | | **P> \|z\|** | | **[95% Conf. Interval]** | |
| Age | | 0.01 | | 0.44 | | -0.01 | 0.03 |
| Education  *(literate compared to illiterate)* | | 0.01 | | 0.93 | | -0.23 | 0.26 |
| Occupation  *(non-farm occupation compared to housewife)* | | -0.04 | | 0.74 | | -0.26 | 0.19 |
| Occupation  *(farm/agriculture compared to housewife)* | | -0.02 | | 0.92 | | -0.32 | 0.29 |
| Proximity to educated people *(yes, no)* | | -0.06 | | 0.62 | | -0.28 | 0.167 |
| Proximity to neighborhood with good welfare *(yes, no)* | | 0.09 | | 0.45 | | -0.14 | 0.32 |
| Transport access *(yes, no)* | | 0.03 | | 0.80 | | -0.22 | 0.29 |
| Walking distance  *(minutes/hours)* | | -0.00 | | 0.07 | | -0.00 | 0.00 |
| Income | | 0.00 | | 0.00 | | 0.00 | 0.00 |
| Urban-rural settings *(urban high-HIV prevalence compared to rural low-HIV prevalence)* | | 0.34 | | 0.01 | | 0.10 | 0.58 |
| Balanced decision-making *(yes, no)* | | -0.05 | | 0.64 | | -0.25 | 0.16 |
| Cons | | 0.46 | | 0.10 | | -0.09 | 1.01 |
| /lnalpha | | -45.946 |  |  |  |  |  |
| Alpha | | 1.11E-20 |  |  |  |  |  |
| Likelihood-ratio test of alpha=0: chibar2(01) = 0.00 Prob>= chibar2 = 1.000 | | | | | | | |
